# Gene expression changes in diapause or quiescent potato cyst nematode, *Globodera pallida*, eggs after hydration or exposure to tomato root diffusate

**DOI:** 10.7717/peerj.1654

**Published:** 2016-02-04

**Authors:** Juan Emilio Palomares-Rius, Pete Hedley, Peter J.A. Cock, Jenny A. Morris, John T. Jones, Vivian C. Blok

**Affiliations:** 1 Institute for Sustainble Agriculture-CSIC, Córdoba, Spain; 2 Cell and Molecular Sciences, The James Hutton Institute, Dundee, United Kingdom; 3 Information and Computational Sciences, The James Hutton Institute, Dundee, United Kingdom; 4 Department of Biology, University of St. Andrews, St Andrews, United Kingdom

**Keywords:** Microarray, *Globodera pallida*, Hatching, Quiescence, Diapause, Gene expression, Nematodes

## Abstract

Plant-parasitic nematodes (PPN) need to be adapted to survive in the absence of a suitable host or in hostile environmental conditions. Various forms of developmental arrest including hatching inhibition and dauer stages are used by PPN in order to survive these conditions and spread to other areas. Potato cyst nematodes (PCN) (*Globodera pallida* and *G. rostochiensis*) are frequently in an anhydrobiotic state, with unhatched nematode persisting for extended periods of time inside the cyst in the absence of the host. This paper shows fundamental changes in the response of quiescent and diapaused eggs of *G. pallida* to hydration and following exposure to tomato root diffusate (RD) using microarray gene expression analysis encompassing a broad set of genes. For the quiescent eggs, 547 genes showed differential expression following hydration vs. hydratation and RD (H-RD) treatment whereas 708 genes showed differential regulation for the diapaused eggs following these treatments. The comparison between hydrated quiescent and diapaused eggs showed marked differences, with 2,380 genes that were differentially regulated compared with 987 genes following H-RD. Hydrated quiescent and diapaused eggs were markedly different indicating differences in adaptation for long-term survival. Transport activity is highly up-regulated following H-RD and few genes were coincident between both kinds of eggs. With the quiescent eggs, the majority of genes were related to ion transport (mainly sodium), while the diapaused eggs showed a major diversity of transporters (amino acid transport, ion transport, acetylcholine or other molecules).

## Introduction

Plant-parasitic nematodes cause damage to crops throughout the world, with costs due to nematode damage calculated as being in excess of US$80 billion each year (Nicol et al., 2011). The potato cyst nematodes (PCN), *Globodera pallida* and *G. rostochiensis*, are important nematode pathogens of potato. Yield losses due to PCN can exceed 50% and there is still a lack of major gene resistance sources for *G. pallida*. This is coupled to the increasing legislative restrictions on the use of nematicides, mean that new control strategies are required (Thorpe et al., 2014).

Some plant pathogens need to be able to survive in the absence of a suitable host and may also need to survive hostile environmental conditions. For cyst nematodes, host range has an important influence on the ability to survive for a prolonged period in the absence of a host, with long-term survival associated primarily with species that have a restricted host range (Perry, 2002). In the case of PCN, cysts with viable eggs can persist for up to 20 years or more in the absence of a suitable host (Lane & Trudgill, 1999). Nematodes that enter this dormant state are frequently desiccated in order to allow them to survive environmental extremes such as freezing. Dormancy is induced by adverse environmental conditions and can be divided into diapause and quiescence. Quiescence is a dormant state that is readily reversible when favourable conditions return, while diapause persists for a set period, even if favourable conditions return (Perry & Moens, 2011). For PCN, diapause is broken after exposure to a period of cold (winter) and is followed by quiescence, which is broken by exposure to root exudates from a suitable host plant. This allows the nematode to ensure that they do not resume their life cycle until winter has passed and a suitable host is growing nearby.

In PCN, development arrest occurs in the second stage juvenile (J2) in the egg before diapause is induced. When diapause is broken hatching of the J2 is activated. The hatching process can be divided into several stages including: (i) changes in eggshell permeability; (ii) activation of the juvenile; and (iii) eclosion (Perry & Moens, 2011). The process starts with a calcium-dependent change in permeability of the lipid layer of the eggshell and subsequent leakage of trehalose out of the egg in response to host root diffusates. Trehalose provides an osmotic stress on the unhatched larva, inducing quiescence and inhibiting locomotion, utilization of energy reserves and also has cryoprotectant activity, giving protection against environmental stresses. Following the leakage of trehalose and subsequent uptake of water, the juvenile then becomes hydrated and increasingly active leading to cutting of the eggshell and hatching. Even in the presence of a host, not all juveniles hatch at the same time; a proportion is retained in the cyst and survives until the next crop (Turner & Rowe, 2006). For this reason a dual model for hatching has been proposed, involving the interaction of specific root diffusates with the eggshell and with the nematode itself (Perry, 1986).

Changes in gene expression that occur during hatching have been characterised using microarrays (Palomares-Rius et al., 2012) and in a life cycle transcriptome analysis undertaken as part of the genome project for *G. pallida* (Cotton et al., 2014). However, little is known about the molecular mechanisms underlying diapause and quiescence in PCN. The aim of this project was to investigate the gene expression changes in diapaused and quiescent eggs following hydration or hydration and exposure to the hatch inducing agent in the root diffusate.

## Material and Methods

### Nematode material

*Globodera pallida* (population Lindley from the James Hutton Institute (JHI) collection) was maintained in glasshouse conditions on the susceptible potato cultivar Désirée. Plants were inoculated with *c*. 5,000 *G. pallida* eggs and maintained in a growth chamber adjusted to 20 ± 1°C, from 60 to 90% relative humidity, and a 14 h photoperiod of fluorescent light of 360 ± 25 µE m^−2^ s^−1^ in a mixture of 2:1 of sand:loam in root-trainers (Ronaash, Kelso, UK). After plants had died, cysts were extracted from the soil by thoroughly mixing infested soil with water in a plastic bucket and settling for 15 s. The supernatant was poured through a 750 µm-pore sieve nested over a 250 µm-pore-sieve. Cysts were collected from the finer mesh sieve and kept at 4°C until used for egg hatching and gene expression experiments.

To prepare tomato root diffusate (RD), roots of 3-week-old tomato plants (cv. Moneymaker) growing in peat were washed, placed in 250 ml of distilled water and kept at room temperature (approximately 20°C) for 2 h. The plant was removed and the water containing root diffusates was filtered through Whatman filter paper and a 0.45 µm syringe filter to remove contaminating micro-organisms. The resulting RD was stored at 4°C until required.

Quiescent cysts and cysts in diapause-quiescence (a percentage of the mixture of cysts could have already passed to quiescence) were obtained from plants grown in 2009 and 2010, respectively, under identical conditions. Cysts from 2009 had been maintained for more than one year at 4°C, to break diapause (Muhammad, 1994). Cysts from populations 2009 (E2009) and 2010 (E2010) were soaked in water for 5 days and subsequently transferred to tomato root diffusate (RD) for 4 days (Fig. S1). These populations are described in Palomares-Rius et al. (2013) and Fig. S1 shows a schematic diagram of how samples were produced. Diapaused-quiescent eggs (E2010) showed a very low percent of hatching in comparison to quiescent eggs (E2009) after a week in RD (10% hatching per 10 cysts, for diapaused-quiescent (E2010) in comparison to quiescent eggs (E2009)) (Palomares-Rius et al., 2013). Eggs were released from cysts using a tissue homogenizer with a clearance of 0.46–0.54 mm between the glass pestle and the homogenizer tube. Cyst walls were removed from eggs by pouring the solution through a 100 µm-pore sieve nested over a 5 µm-pore-sieve. Eggs were concentrated by decantation and centrifugation.

### Microarray design and annotation

#### Microarray design

A custom microarray was designed based on a pre-publication draft of the *G. pallida* genome annotation (Cotton et al., 2014), supplemented with probes used in an older array based on EST sequences (Palomares-Rius et al., 2012). Up to five probes per annotated gene were designed with Agilent eArray (Agilent Technologies, Santa Clara, CA, USA) using the “Base Composition Methodology” for sense probes between 40 and 60 bp, with a 3′ bias. Separately, Array Designer 4 (PREMIER Biosoft International, Palo Alto, CA, USA) was used to generate seven sets of probes trading specificity against probe length and melting temperature. The combined candidate probe list was screened against all the target sequences using BLASTN forward matches only to verify the matches and exclude potentially cross hybridising probes. Redundant probes where one was a subsequence of another were removed. The first three probes per sequence target were selected preferring the eArray probes and then the most specific Array Designer probes. Combined with probes used in Palomares-Rius et al. (2012) not matching this version of the annotation, this gave 59,509 probes from 20,834 target sequences (annotated genes, ESTs, or genes in GenBank). These were uploaded to Agilent eArray, and randomly laid out on the array using the default Agilent linker used to extend the 3′ end of the shorter probes to length 60 bp.

#### Microarray annotation

The *G. pallida* genome was published after the array was designed, and therefore the probe sequences were re-mapped to the revised the genome annotation. Basic re-annotation of the 16,417 matched protein sequences from the *G. pallida* genome was performed using the JHI installation of Galaxy (Giardine et al., 2005). The NCBI NR database was searched with BLASTP (from NCBI BLAST+ version 2.2.26, with *e*-value cut-off 0.001) (Camacho et al., 2009), and then processed using Blast2GO (Conesa et al., 2005) for pipelines (b2g4pipe) v2.5 running against a local Blast2GO database from May 2011 (Cock et al., 2015). SignalP v3 (Bendtsen et al., 2004; Nielsen & Krogh, 1998) and TMHMM v2 (Krogh et al., 2001) were also used in Galaxy in order to identify secreted proteins, including candidate effectors (Cock et al., 2013).

The annotations allowed information about putative function to be applied to 35.3% of the targeted gene sequences (7,355 of 20,834 genes). The 2,025 secreted proteins (genes with a predicted signal peptide and lacking a transmembrane domain) were identified in the genes represented on this microarray.

### RNA extraction and microarray hybridisation

Total RNA was extracted using an RNeasy^®^ Plus Micro Kit (Qiagen, Hilden, Germany) following the manufacturer’s instructions. DNA digestion was conducted on column during RNA extraction using RNase-Free DNase set (Qiagen, Hilden, Germany) as recommended. Total RNA was quantified using a 2100 Bioanalyzer (Agilent Technologies) following the manufacturer’s instructions.

In total, 12 hybridisations for each microarray experiment were performed (detailed in ArrayExpress accession E-MTAB-3824along with all raw data sets). Microarrays were hybridised and scanned by the Genome Technology group at the JHI using standard procedures. Approximately 10–20 ng of each total RNA extraction was used for RNA amplification and hybridization. Labelling of total RNA was carried out using the Low Input Quick Amp Labelling Kit (Agilent Technologies) as recommended. All microarray hybridisation and washing procedures were performed according to the Two-Color Microarray-Based Gene Expression Analysis guide (v.5.7; Agilent Technologies). Microarrays were imaged using a G2505B scanner (Agilent Technologies, Santa Clara, CA, USA) at 5 µm resolution with extended dynamic range (XDR).

### Microarray data analysis

Data were extracted from microarray images using Feature Extraction software (v.9.5; Agilent Technologies) before being imported into Genespring (v.7.3; Agilent Technologies, Santa Clara, CA, USA) for QC and data analysis. Normalisation of raw data was performed using the Lowess algorithm. Data sets were filtered to remove non-reliable data (intensity values ≥50 in 4/16 samples). Data was log transformed prior to statistical analysis. All the statistical and CLICK analysis were performed with the program Expander v.6.06 (Ulitsky et al., 2010) and standard statistical tests were applied to identify significant differential gene expression. A Student’s *T*-test was used with *p*-value cutoff ≤0.01, and a minimum fold change cut-off ≥2 between tested samples and taking the mean of all designed probes for the specified gene. Fisher enrichment test (FDR < 0.05) was performed in Blast2GO v.2.7.0 for the different functional comparisons in the studied microarray samples.

### Real-time (RT-PCR)

Six genes differentially expressed respective to their different life-stages or host genotypes and one gene without differential expression were validated, along with three housekeeping reference genes (see Table 1). Housekeeping genes were chosen according their consistent stable expression across all samples in the microarray. Primer design was conducted on-line using the Primer3 program (Rozen & Skaletsky, 2000).

**Table 1:** RT-PCR validation using 3 housekeeping and 6 differentially expressed genes in quiescent hydrated and hydrated-RD *Globodera pallida* eggs.

				Stages ^*^	
Probes	Gene	Primers	BLAST hit	M	Q
CUST_3830,	GPLIN_000489300	F: 5′CCCATAAGTGCCCAATTTGT3′	No match	2.90	2.1
CUST_3831,		R: 5′TCATCTTTGGCTGCAGAATG3′			
CUST_3832					
CUST_40764,	GPLIN_000340000	F: 5′CCAAACCCTTCGAAGATCAA3′	dimethylaniline monooxygenase	6.43	2.92
CUST_40765,		R: 5′GCCACTTTGCTAAGCTCCAC3′			
CUST_40766					
CUST_34634,	GPLIN_001125100	F: 5′GAGATGCTCCCATCGACTGT3′	ph domain-containing protein 2	2.50	1.95
CUST_34635,		R: 5′GCGTGTAATGGGAGCTGAAT3′			
CUST_34636					
CUST_684,	GPLIN_001330000	F: 5′TTCAATTCCTTTTCCGGATG3′	ras guanyl-releasing protein 3	2.43	1.7
CUST_685,		R: 5′TGCCATCAGCGTGTTAAAAG3′			
CUST_686					
CUST_15290,	GPLIN_000723500	F: 5′TGACATTGAGCCTGAACTGC3′	haloacid dehalogenase subfamily variant 3 with third motif having dd or ed	6.14	8.57
CUST_15291,		R: 5′TGCCAGAAGAACGGAGAGAT3′			
CUST_15292					
probe003464,	GPLIN_000127800	F: 5′AATGAGCATTCCGAGTGGAC3′	No match	2.76	2.1
probe003466,		R: 5′TGGTCTTTTGTCCGGGTTAG3′						
probe006774,					
probe006776,					
CUST_13407,					
CUST_13408,					
CUST_13409					
Housekeeping genes^**^					
CUST_47827,	GPLIN_000606600	F: 5′CACACAGATGGGGTGAGATG3′	mitotic checkpoint protein bub3	–	–
CUST_47828,		R: 5′GTACACTCTGTCCCCGCATT3′			
CUST_47829					
probe004575,	GPLIN_000738000	F: 5′CCGCTGGACTTGTTGGTAAT3′	anaphase-promoting complex subunit 1	–	–
probe004576,		R: 5′TAAGCTGCAGCACATCCAAC3′			
probe009599,					
CUST_15991,					
CUST_15992,					
CUST_15993					
CUST_25109,	GPLIN_000931300	F: 5′GCCAATGTGTTGATCACAGG3′	No match	–	–
CUST_25110,		R: 5′TCGGTACCACAAAGTGACCA3′			
CUST_25111					

**Notes.**

* M, microarray; Q, QRT-PCR.

** Genes with expression not significantly modified in the microarray analysis after ANOVA on genes which passed the filtering criteria, with *p*-value cutoff ≤0.05 and Bonferroni multiple testing correction and taking the average of all designed probes for the specified gene.

Total RNA from a replicated experiment was extracted using the RNeasy^®^ Plus Micro Kit (Qiagen, Hilden, Germany) following the manufacturer’s instructions. DNA digestion was conducted during RNA extraction using an RNase-Free DNase set (Qiagen, Hilden, Germany) following the manufacturer’s instructions. Approximately 700 ng of total RNA was used for cDNA synthesis using QuantiTect^®^ Reverse Transcription Kit (Qiagen, Hilden, Germany) following the manufacturer’s instructions. The cDNA was diluted 1:20 with PCR grade water and then used in real-time PCR reactions containing iQ™ SYBR^®^ Green Supermix (Bio-rad, Hercules, CA, USA) with a primer concentration of 200 mM each and 5 µl of the diluted cDNA in a final volume of 15 µl. Cycling conditions consisted of one cycle of denaturing at 94°C for 3 min followed by 40 cycles of 15 s denaturing at 94°C, 30 s annealing at 55°C and 30 s extension at 72°C, finishing with an final extension of 5 min at 72°C. All real-time PCR assays were performed using a Chromo 4™ Real-Time Detector (Bio-RAD, Hercules, CA, USA). PCR products obtained from the different extractions were verified by performing a melting curve analysis and visualization of PCR products on 1.5% agarose gels stained with SYBR^®^ Safe DNA gel stain (Invitrogen Corp., Carlsbad, CA, USA) following the manufacturer’s instructions in order to verify the size of the product obtained in the different extractions. All samples were run in triplicate and results were analyzed using Gene Expression Macro™ ver. 1.1 (Bio-Rad, Hercules, CA, USA), software provided by the manufacturer.

## Results and Discussion

### Genes regulated during the hatching process in quiescent and diapaused eggs and between hydrated and hydrated-RD soaked eggs

The hatching process of *G. pallida* eggs was studied in four comparisons. A diagram of the samples is shown in Fig. S1 and annotated genes used in the microarray are in Table S1. Comparisons were as follows: (i) hydrated *vs*. hydrated-Root diffusate (H-RD) quiescent eggs (population E-2009); (ii) hydrated *vs*. H-RD soaked diapaused eggs (population E-2010); (iii) hydrated quiescent *vs*. hydrated diapaused eggs (E2009 *vs*. E2010, respectively); (iv) H-RD quiescent *vs*. diapaused eggs (E2009 *vs*. E2010, respectively). The first and second comparisons were aimed at finding genes responding during the hatching process and the third and fourth comparisons were performed in order to find genes related to hatching inhibition and survival during the hatching process (H-RD treatment) or already expressed before the hatching activation (water treatment). The diapaused juveniles population (E2010) could be defined as a mixture of a majority of eggs in diapause stage and a minor portion of quiescent juveniles (approximately 10% of the total egg population) because this same population had a proportion of juveniles hatched when exposed to RD (Palomares-Rius et al., 2012). A Student’s *T*-test (*P* ≤ 0.01) was used to identify genes with significantly changes of at least two-fold expression differences.

For the quiescent eggs, 547 genes showed differential expression following hydration *vs*. H-RD treatments (Table S2); 368 and 179 were up-regulated and down-regulated, respectively. In contrast, 708 genes showed differential regulation for the diapaused eggs following hydration *vs*. H-RD treatments (Table S3); 523 and 185 genes were up-regulated and down-regulated, respectively. The comparison between hydrated quiescent and diapaused eggs showed that there were 2,380 genes differentially regulated; 760 and 1,588 genes were up-regulated and down-regulated, respectively (Table S4). The comparison between H-RD soaked quiescent and diapaused eggs showed that 987 genes were differentially regulated; 245 and 742 genes were up-regulated and down-regulated, respectively (Table S5).

**Figure 1:**
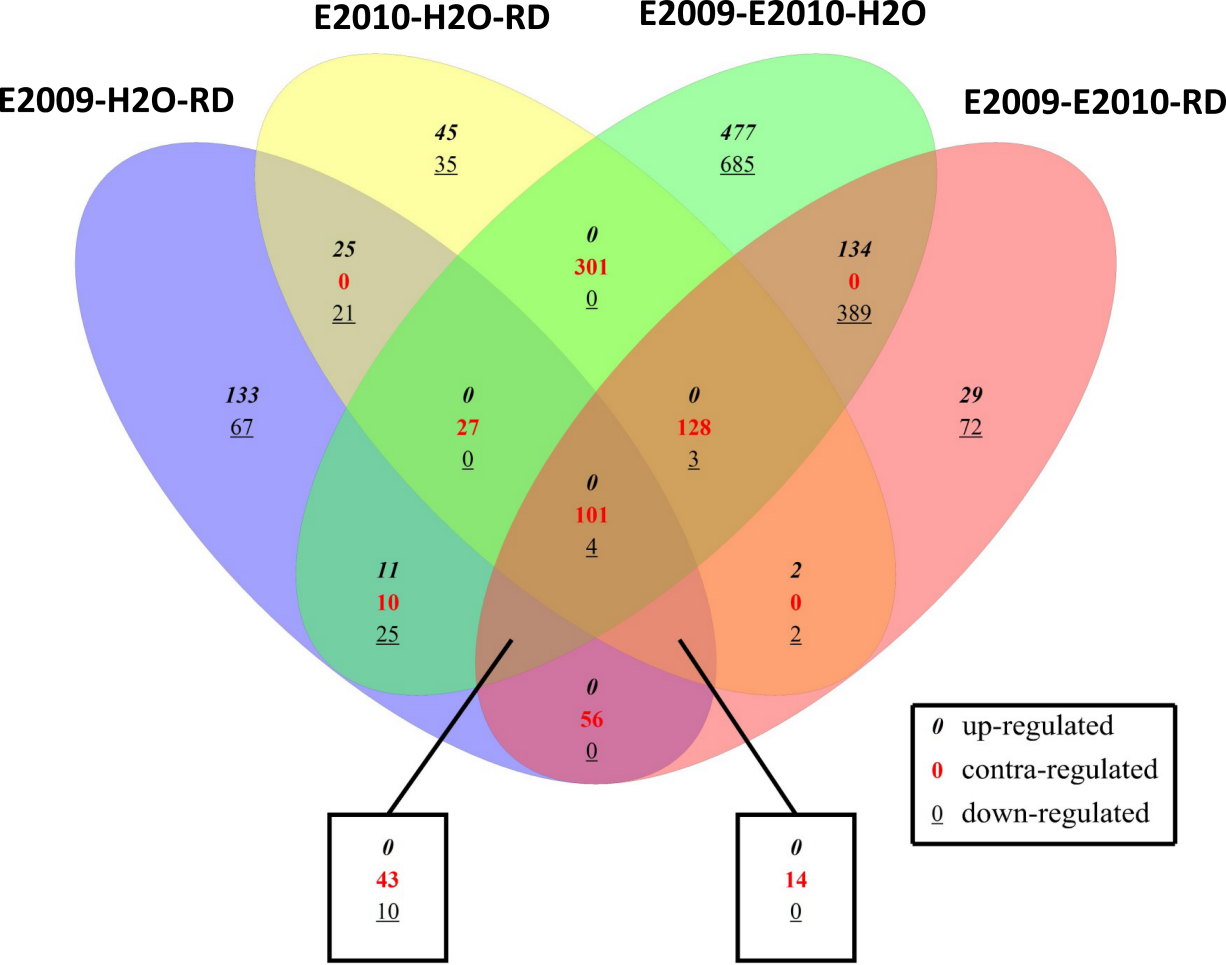
Venn diagram for genes differentially expressed of Globodera pallida between the different comparisons. E2009-H2O-RD, comparison between hydrated and hydrated-RD soaked quiescent eggs; E2010-H2O-RD, comparison between hydrated and hydrated-RD soaked diapaused eggs; E2009-E2010-H2O, comparison between hydrated quiescent and diapaused eggs; E2009-E2010-RD, comparison between hydrated-RD soaked quiescent and diapaused eggs.

A Venn diagram of genes differentially expressed in each comparison of quiescent or diapaused eggs following hydration or H-RD treatments is presented in Fig. 1 (Figs. S2 and S3). Interestingly, some genes are unique and differentially expressed in certain comparisons. For example, 355 genes (222 and 133 were up-regulated and down-regulated, respectively) were uniquely differentially expressed in hydrated compared to H-RD soaked quiescent eggs and 516 genes (377 and 139 were up-regulated and down-regulated, respectively) were uniquely differentially expressed in hydrated compared to H-RD soaked diapaused eggs. In comparison, 144, 44 and 4 genes were up-regulated, down-regulated and contra-regulated (expressed in different level sense between treatments), respectively in both hydrated quiescent and diapaused eggs or compared to H-RD soaked quiescent and diapaused eggs (Tables S6–S8), while hydrated quiescent and diapaused eggs had 1,536 genes in common (557 and 979 genes up-regulated and down-regulated respectively). H-RD soaked quiescent and diapaused egg shared 175 genes in common (45 up-regulated and 130 down-regulated). Although many genes were in common in the comparisons, the majority of them are contra-regulated (differentially up-regulated or down-regulated, depending on the treatment); 200, 3 and 609 were in common that were up-regulated, down-regulated and contra-regulated, respectively in hydrated quiescent and diapaused eggs compared with these eggs following H-RD soaking (Tables S9–S11).

**Figure 2:**
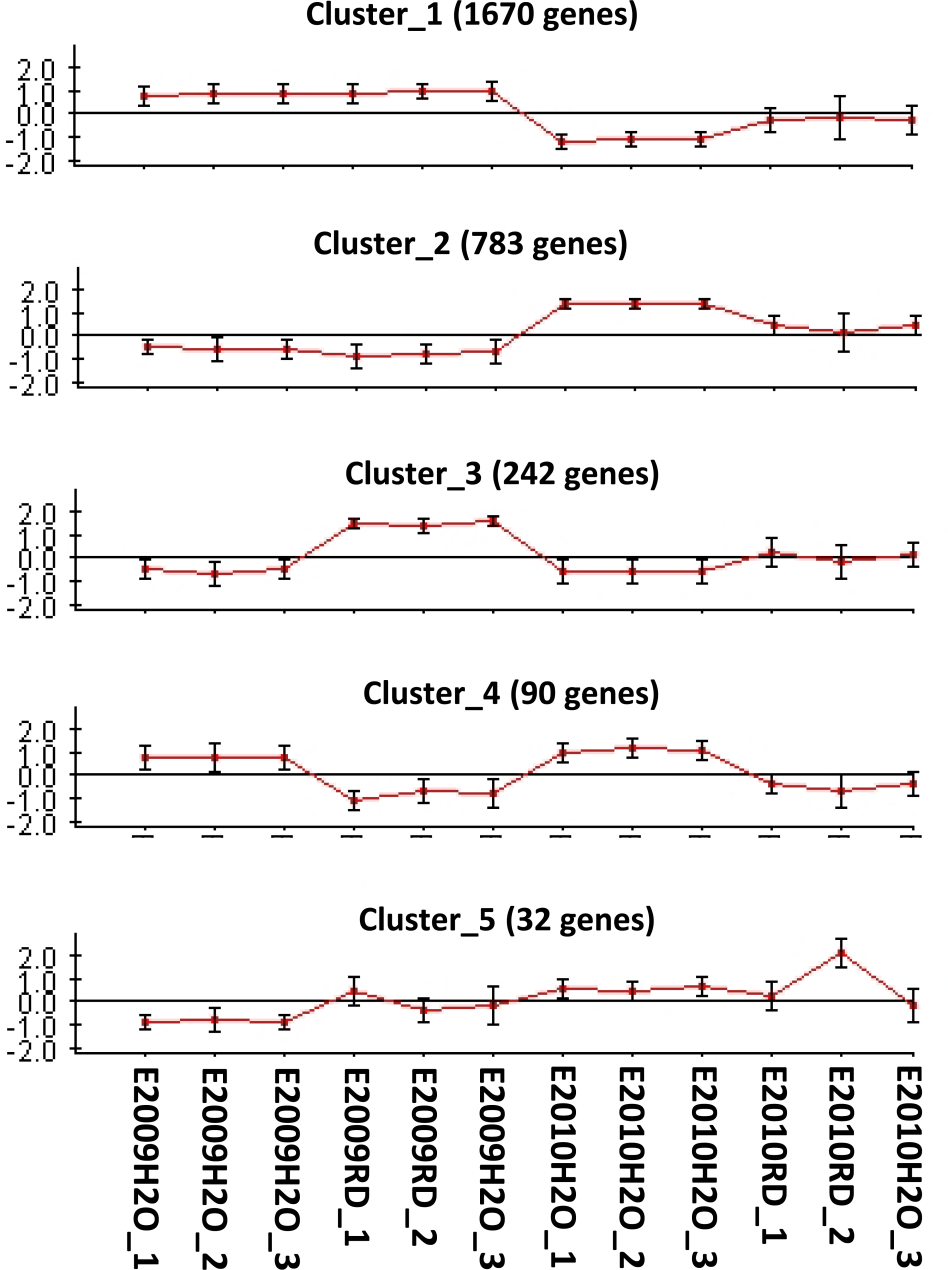
CLICK clustering of differentially expressed genes of Globodera pallida. E2009H2O: hydrated quiescent eggs; E2010H2O: hydrated diapaused eggs; E2009RD: hydrated-RD soaked quiescent eggs; E2010RD: hydrated-RD soaked diapaused.

Genes that were up-regulated in both quiescent and diapaused eggs after H-RD treatment encode collagen-related proteins, aquaporin, transporters, glycosyl hydrolases, annexins, peptidases and metabolic enzymes (Table 2 and Table S6). Some of these genes could be related to internal organ remodelling because of the abundance of peptidases that are not secreted (Table 1) (Blelloch & Kimble, 1999). Another protein related to body remodelling is a Zinc finger protein c3h1 type-like 1 (GPLIN_001152100), that belongs to the ZFP36 family of RNA-binding proteins, which contains two characteristic tandem CCCH-type zinc-finger domains (Xia, Zhao & Mao, 2012). Xenopus zinc finger protein 36, C3H type-like 1, is involved in neural development and might play an important role in post-transcriptional regulation (Xia, Zhao & Mao, 2012).

**Table 2:** Examples of genes expressed in both hydrated and hydrated-RD soaked quiescent and diapaused *Globodera pallida* eggs (E2009 and E2010). Sequence descriptions were obtained using Blast2Go (v.2.7.0). More information is in Tables S6 and S7. RD, Tomato root diffusate. (Not including transport function proteins.)

Seq. names	Seq. description	InterproScan
Up-regulated		
GPLIN_000915600	amidinotransferase family protein	no IPS match
GPLIN_000894700; GPLIN_000886200	annexin	no IPS match- TMHMM
GPLIN_000876700	cathepsin l-like cysteine proteinase	no IPS match
GPLIN_000778700	cuticle collagen	TMHMM
GPLIN_001473100; GPLIN_000242800	fatty acid elongation protein 3	TMHMM
GPLIN_001036800	membrane metallo-endopeptidase-like 1-like	no IPS match
GPLIN_000461500; gi|54547695|gb|CV578335.1| CV578335	n-acetylated-alpha-linked acidic dipeptidase 2	no IPS match
Gpa_EST_04_1___F07_027	o-glycosyl hydrolase family 30 protein	no IPS match
gi|54548456|gb|CV578708.1|CV578708; GPLIN_000440000	peptidase c13 family protein	
GPLIN_001529500; GPLIN_000655800	peptidase family m13	no IPS match
GPLIN_000575100	protein aqp- isoform b (aquaporin)	TMHMM
GPLIN_001152100	zinc finger protein c3h1 type-like 1	SignalP-TM
Down-regulated		
GPLIN_001294200	adipocyte plasma membrane-associated protein	no IPS match
GPLIN_001345700; GPLIN_000827200	beta -endoglucanase	SignalP-noTM
GPLIN_000936300	glutathione synthetase-like	no IPS match
GPLIN_000784100	myosin (hum-6)	no IPS match
GPLIN_000297000	nuclear receptor nhr-61	no IPS match
GPLIN_000408000	ring-h2 finger protein	no IPS match
GPLIN_000693300	serine-type endopeptidase (try-1)	TMHMM

Common genes down-regulated in both quiescent and diapaused eggs after H-RD treatment encode endo-glucanases, a signal receptor, transcription factors and endopeptidases genes (Table 2 and Table S7). Surprisingly, some beta-endoglucanase genes were down-regulated in both kinds of eggs even when they seem to be expressed in infective nematodes and are required for penetration and migration through plant tissues (Chen et al., 2005). Only one gene was identified on the basis of BLAST2GO (methylthioribulose-1-phosphate dehydratase (GPLIN_000485200)) in the contra-regulated genes (Table S8).

More genes were differentially expressed than in common for both types of eggs (Table 3 and Tables S12–S15) following H-RD treatment. Surprisingly, we observed many genes encoding putative effectors or digestive enzymes that were expressed in the diapaused eggs, including arabinogalactan endo-beta-galactosidase (Vanholme et al., 2009), beta-endoglucanase, cathepsin (Urwin et al., 1997), expansin (Qin et al., 2004), pectate lyase 2 (Kudla et al., 2007) and venom allergen-like protein (Lu, Yu & Wang, 2007). One possible explanation is that these genes could have multiple copies in the *G. pallida* genome (Cotton et al., 2014) with different expression patterns. For example, specific copies of the venom allergen-like protein gene have different expression patterns in *M. hispanica* eggs (Duarte et al., 2014). Another explanation could be that a proportion of the eggs are sensitive to the RD and are in the process of hatching (quiescent eggs), even in the population where the majority is composed of diapaused eggs. Galactoside-binding lectin family protein (GPLIN_000170000) and glutathione s-transferase (several, see Table 2) genes were down-regulated in quiescent eggs before hatching.

**Table 3:** Selected genes uniquely differentially expressed between hydrated and hydrated-RD soaked quiescent and diapaused Globodera pallida eggs (E2009 and E2010). Sequence descriptions were obtained using Blast2Go (v.2.7.0). More information is in Tables S12 and S13. (Not including transport function protein.) RD, Tomato root diffusate.

Seq. names	Seq. description	Seq. names	Seq. description
Up-regulated unique E2009		Up-regulated unique E2010	
GPLIN_000972400	annexin a7	GPLIN_000142900; GPLIN_000143000	arabinogalactan endo–beta-galactosidase
GPLIN_000409100	aspartic protease	GPLIN_001215600; GPLIN_001111300; GPLIN_001111200; GPLIN_000536400; GPLIN_000933200; GPLIN_000755200; GPLIN_000412500; GPLIN_000616300; GPLIN_001007400	beta-endoglucanase
Gpa_EST_P1_02_01_C4_C04_014; GPLIN_001099500	cathepsin l-like cysteine proteinase	Contig523; GPLIN_000196600; GPLIN_001613900; Contig251	cathepsin
GPLIN_001532300; GPLIN_000183600	chitinase	GPLIN_000747800; GPLIN_000107100	collagen
Contig254	collagen	rc_J2contig298	expansin
GPLIN_000340000	dimethylaniline monooxygenase	GPLIN_001093500	galactoside-binding lectin family protein
GPLIN_001237300; GPLIN_000080000; GPLIN_001502700; Contig491; Contig502	heat shock protein 70	GPLIN_001153000	glutathione peroxidase
GPLIN_001595700; GPLIN_000887800; Gpa_Est_04_02_B6_B06_023	heat shock protein 90	Contig564; GPLIN_000504600; gi|54549230|gb|CV579086.1|CV579086	heat shock protein 70
GPLIN_001206900	n-acetylated-alpha-linked acidic dipeptidase 2	GPLIN_000142600	pectate lyase 2
GPLIN_001557000	tau-tubulin kinase 1	Contig751; GPLIN_000653700	thioredoxin peroxidase
GPLIN_001424300	zinc finger protein	rc_J2contig172; GPLIN_001139400; GPLIN_000445700	venom allergen-like protein
Down-regulated unique E2009		Down-regulated unique E-2010	
GPLIN_000170000; rc_Gpa_EST_07_3___E09_036	galactoside-binding lectin family protein	GPLIN_000960100; GPLIN_001638400	cuticle collagen
GPLIN_001198500; GPLIN_000240500; GPLIN_000030600	glutathione s-transferase-1	GPLIN_000404900	glutathione synthetase
GPLIN_000706500	glutathione synthetase	GPLIN_000768300	malate dehydrogenase
GPLIN_000552100	glycerol kinase	GPLIN_000327200	nuclear hormone receptor family member nhr-14
GPLIN_001404500	thaumatin-like protein	GPLIN_000475300	s- glutathione dehydrogenase

### Functional annotation of genes involved in hatching and in quiescence/ diapause

Gene annotation comparisons between quiescent and diapaused eggs exposed to H-RD showed some common molecular functions (binding, catalytic, molecular transducer, nucleic acid binding transcription factor and receptor activities) in the genes that were up-regulated or down-regulated (Fig. S3). Others were different only for up-regulated genes (antioxidant, electron carrier, enzyme regulator, and transporter activities). Transporter activity in both cases was highly activated in both egg types. Comparison of the molecular functions between quiescent and diapaused hydrated eggs showed similar patterns in the genes that were up-regulated and down-regulated (antioxidant, binding, catalytic, enzyme regulator, molecular transducer, nucleic acid binding transcription factor, receptor, structural molecule and transporter activities), while electron carrier activity was only down-regulated in diapaused eggs. Comparison of molecular functions between quiescent and diapaused eggs soaked in H-RD showed similar functions (antioxidant, binding, catalytic, molecule transducer, nucleic acid binding transcription factor and structural activities), but electron carrier, receptor and transporter activity genes were specifically down-regulated in diapaused eggs.

Similar biological processes were affected (Fig. S2) for genes up-regulated or down-regulated (biological adhesion, biological regulation, cellular component organization or biogenesis, cellular process, developmental process, growth, immune system process, localization, locomotion, metabolic process, multi-organism process, multicellular organismal process, reproduction, response to stimulus, rhythmic process, signalling, single-organism process). The immune system process was not affected in the comparisons between quiescent or diapaused eggs that were H-RD treated.

### Transport activity is highly up-regulated in eggs soaked in RD

Transporter activity was studied in detail because of the significant increase when both types of eggs were soaked in RD. In total, 21 and 29 transporter activity genes were significantly up-regulated in the H-RD treated quiescent and diapaused eggs, respectively (Table 4). The majority of the encoded proteins had a predicted transmembrane domain and are also related to ion transport and the nervous system and were different between both kinds of eggs; only four genes were up-regulated in both egg types. These genes include aquaporin (GPLIN_000575100), proton dependent oligopeptide (POT) transporter (GPLIN_000878400), two-P domain potassium channel (GPLIN_000894900), ion transporter (GPLIN_001017500 and GPLIN_001269400). With the quiescent eggs, the majority of genes were related to ion transport (mainly sodium), while the diapaused eggs showed a major diversity of transporters (amino acid transport, ion transport, acetylcholine or other molecules).

**Table 4:** Genes differentially expressed between hydrated and hydrated-RD soaked quiescent or diapaused *Globodera pallida* eggs (E2009 and E2010, respectively) related to transport molecular functions. Sequence descriptions and InterProScan is obtained using Blast2Go (v.2.7.0). RD, Tomato root diffusate.

Seq. name	Fold change E2009H2O vs. E2009RD	Fold change E2010H2O vs. E2010RD	Seq. length	Seq. description	InterProScan
GPLIN_000661700	2,35	–	80	b(+)-type amino acid transporter 1	TMHMM
GPLIN_001367300	2,82	–	707	protein clh- isoform a	TMHMM
GPLIN_001554300	3,09	–	244	protein nac- isoform a	TMHMM
GPLIN_000595000	3,42	–	311	protein nhx- isoform a	TMHMM
GPLIN_000832100	4,02	–	734	protein nhx- isoform b	TMHMM
GPLIN_001216000	2,02	–	213	sarco endoplasmic reticulum calcium atpase	TMHMM
GPLIN_001285100	2,27	–	402	sideroflexin 3	TMHMM
GPLIN_001225100	2,77	–	866	sodium- and chloride-dependent neurotransmitter transporter	TMHMM
GPLIN_000057300	2,21	–	1,269	sodium bicarbonate transporter-like protein 11	TMHMM
GPLIN_001536500	2,06	–	176	sodium bicarbonate transporter-like protein 11	TMHMM
GPLIN_000467600	3,06	–	243	sodium:neurotransmitter symporter family protein	TMHMM
GPLIN_000278200	3,17	–	457	solute carrier family 13 member 2	TMHMM
GPLIN_001105900	3,26	–	560	solute carrier family 13 member 5	TMHMM
GPLIN_000288600	2,01	–	305	synaptotagmin 1-like	no IPS match
GPLIN_000966100	2,74	–	749	two-component system sensor protein	TMHMM
GPLIN_000899700	–	2,32	630	acr-17-like protein	TMHMM
GPLIN_000440700	–	2,45	496	amino acid permease	TMHMM
Contig721	–	2,3	525	atp synthase beta	no IPS match
GPLIN_000483900	–	2,1	538	atp synthase subunit mitochondrial	SignalP-TM (SIGNALP_GRAM_POSITIVE)
GPLIN_000343300	–	2,35	218	cre-aat-1 protein	TMHMM
GPLIN_000460800	–	2,09	907	cre-twk-29 protein	TMHMM
GPLIN_000125500	–	2,68	497	cre-unc-58 protein	TMHMM
GPLIN_000429500	–	3,66	513	excitatory amino acid transporter 2-like	TMHMM
GPLIN_000897100	–	2,73	701	glutamate gated chloride channel alpha 3	TMHMM
GPLIN_001045400	–	3,13	593	high-affinity choline transporter 1	TMHMM
GPLIN_000949400	–	2,29	909	hydroxyacid-oxoacid mitochondrial	TMHMM
GPLIN_000353800	–	2,29	142	karyopherin alpha 4	no IPS match
GPLIN_000940400	–	4,09	466	protein del- isoform a	TMHMM
GPLIN_000516300	–	2,16	395	protein egl- isoform b	TMHMM
GPLIN_001385100	–	2,09	832	protein haf- isoform a	TMHMM
Contig621	–	2,32	960	protein isoform c	no IPS match
GPLIN_000618200	–	2,08	621	protein lgc- isoform a	SignalP-noTM; TMHMM
GPLIN_001195300	–	2,23	549	protein lgc- isoform b	SignalP-noTM; TMHMM
GPLIN_000846800	–	3,18	235	protein lgc-38	TMHMM
GPLIN_000376100	–	2,1	536	protein snt- isoform a	TMHMM
GPLIN_000591700	–	4,86	375	solute carrier family facilitated glucose transporter member 3-like	TMHMM
GPLIN_000261800	–	2,24	492	twik (kcnk-like) family of potasium alpha subunit 40	TMHMM
GPLIN_001540600	–	2,05	170	uncoordinated protein 58	TMHMM
GPLIN_000688100	–	3,37	518	vesicular acetylcholine transporter unc-17	TMHMM
GPLIN_000575100	6,85	7,42	283	protein aqp- isoform b	TMHMM
GPLIN_000878400	4,49	2,24	207	pot family protein	TMHMM
GPLIN_000894900	2,84	4,2	631	protein twk- isoform a	TMHMM
GPLIN_001017500	2,06	3,22	406	ion transport protein	SignalP-TM; TMHMM
GPLIN_001269400	4,22	2,49	230	protein nhx- isoform a	no IPS match

More membrane transporter genes were activated in the diapaused than in the quiescent eggs, however, this could be related to the sampling point (4 days soaking in RD), as expression in quiescent eggs may have already dissipated by this time. The strong hatching response of quiescent eggs following stimulation with RD could also correspond to a more synchronised expression of particular genes than in the diapaused eggs, which may have more prolonged gene activation.

Aquaporin channels facilitate the transport of water, glycerol, and other small solutes across cell membranes (Huang et al., 2007). Juvenile hydration in *G. rostochiensis* has been observed during the hatching process: the egg loses trehalose from the perivitelline fluid after exposure to host RD and even after hatching (Jones, Tylka & Perry, 1998). How this is involved in diapaused eggs is difficult to explain, because the general hatching model only takes into account the trehalose release in a Ca^2+^-mediated eggshell permeability after hatch stimulation with host RD (Jones, Tylka & Perry, 1998). One gene coding a NHX isoform protein was up-regulated in both types of eggs treated with H-RD. This family belongs to the Na^+^–H^+^ exchangers. These proteins prevent cellular acidification by catalyzing the electroneutral exchange of extracellular sodium for an intracellular proton (Nehrke & Melvin, 2002). They are involved in cell volume regulation, fluid secretion and absorption, and pH homeostasis (Nehrke, 2003). Transcripts of different *nhx* gene are distributed in different parts of the body in *C. elegans* (Nehrke & Melvin, 2002) and these genes could be involved in processes that occur before hatching, consistent with higher expression in hydrated quiescent compared to diapaused eggs. A pot family protein (GPLIN_000878400) was also up-regulated in both egg types (at higher levels in quiescent eggs). These proteins are proton-dependent oligopeptide transporters and their role seems related to short chain peptide absorption in body re-modelling (Solcan et al., 2012).

Several genes potentially involved in the process of neurotransmission showed different expression profiles in diapaused and quiescent eggs in response to RD. Quiescent eggs had a high number of protein transporters related to sodium, whereas potassium neurotransmitters were associated with the diapaused eggs. Neural excitability and neurotransmitter release has been related to potassium channels and they are well represented in the genome of *C. elegans* (Bargmann, 1999; Gu & Barry, 2010). Sodium symporters of the plasma membrane mediate the cellular uptake of neurotransmitters from the synaptic cleft (Ribeiro & Patocka, 2013). These transporters control how much transmitter is released and how long it remains in the synaptic cleft, thereby regulating the intensity and duration of signalling neurotransmitters (Ribeiro & Patocka, 2013). These two important and quite antagonistic functions with different genes and isoforms of the same gene could show a “ready mode” for quiescent eggs, while the diapaused eggs are primed, but not yet “ready” for hatching. A further possibility is that different types of neurons each express a distinct set of channel proteins and target them to a different set of subcellular compartments or distribution in the cell (Gu & Barry, 2010). Additionally, the expansion of potassium transporters in the *C. elegans* genome has been proposed to be related to their expression in other cell types, in addition to neurons (Salkoff et al., 2005). The protein TWK (two-P domain K + channel) (GPLIN_000894900) could be a common regulator of the neurosystem in both kinds of eggs. This type of potassium channel is much expanded in the genome of *C. elegans* and it has been suggested that it may reflect a special adaptation of *C. elegans* to customize the electrical properties of individual cell types (Salkoff et al., 2001; Salkoff et al., 2005).

Other genes differentially up-regulated included the mitochondrial ATP synthase subunit (GPLIN_000483900); mitochondrial protein hydroxyacid-oxoacid transhydrogenase (GPLIN_000949400) and the protein HAF (GPLIN_001385100). Protein HAF (hypoxia associated factor) is a member of the ABC transporter family with high expression in tissue with rapid growth (Koh & Powis, 2009). This suggests that increasing metabolic changes are associated with the diapaused eggs.

### Biological functions enriched and genes regulated during the hatching process in quiescent and diapaused eggs

Several functions were enriched in the different comparisons studied and are summarized in Table 5. Quiescent eggs treated with H-RD showed enrichment in secondary active transmembrane transporter activity, while no functions were enriched in the down-regulated genes. The soaking of diapaused eggs in RD produced enrichment in many functions related to molecule transport, receptor activity, carbohydrate binding and hydrolase activity and alcohol dehydrogenase (NAD) activity while no functions were enriched in the down-regulated genes. Quiescent and diapaused eggs soaked in water did not show any enrichment in the up regulated genes. The expression of transport, hydrolase, polysaccharide, DNA and RNA binding, kinase activity and galactosidase activities were more enriched in the down-regulated genes.

**Table 5:** Fisher enrichment test (FDR < 0.05) performed in Blast2GO v.2.7.0 for the different comparisons of hydrated and RD-hydrated quiescent and diapaused eggs of *Globodera pallida* in the microarray study (E2009 and E2010, respectively). RD, Tomato root diffusate.

GO-ID	Term	*P*-value	FDR
**E2009-H2O** **vs.** **E2010-RD (up-regulated)**			
GO:0015291	secondary active transmembrane transporter activity	2.78E−7	2.19E−3
**E2009-H2O vs. E2009-RD (down-regulated)**			
–	–	–	–
**E2010-H2O** **vs.** **E2010-RD (up-regulated)**			
GO:0008810	cellulase activity	9.74E−12	7.68E−8
GO:0005215	transporter activity	1.16E−5	5.46E−3
GO:0004550	nucleoside diphosphate kinase activity	1.88E−5	6.75E−3
GO:0003676	nucleic acid binding	2.99E−5	9.44E−3
GO:0004022	alcohol dehydrogenase (NAD) activity	3.55E−4	4.99E−2
**E2010-H2O** **vs.** **E2010-RD (down-regulated)**			
–	–	–	–
**E2009-H2O** **vs.** **E2010-H2O (up-regulated)**			
–	–	–	–
**E2009-H2O** **vs.** **E2010-H2O (down-regulated)**			
GO:0004553	hydrolase activity. hydrolyzing O-glycosyl compounds	2.35E−15	4.63E−12
GO:0022891	substrate-specific transmembrane transporter activity	2.75E−11	1.67E−8
GO:0003676	nucleic acid binding	7.01E−11	3.25E−8
GO:0008810	cellulase activity	7.95E−11	3.48E−8
GO:0097159	organic cyclic compound binding	1.10E−10	4.09E−8
GO:1901363	heterocyclic compound binding	1.11E−10	4.09E−8
GO:0022803	passive transmembrane transporter activity	1.24E−10	4.09E−8
GO:0015267	channel activity	1.24E−10	4.09E−8
GO:0016917	GABA receptor activity	1.22E−5	1.46E−3
GO:0015077	monovalent inorganic cation transmembrane transporter activity	1.39E−5	1.63E−3
GO:0043499	eukaryotic cell surface binding	5.82E−5	5.40E−3
GO:0003723	RNA binding	1.83E−4	1.46E−2
GO:0004563	beta-N-acetylhexosaminidase activity	2.07E−4	1.47E−2
GO:0004550	nucleoside diphosphate kinase activity	2.07E−4	1.47E−2
GO:0003677	DNA binding	4.41E−4	2.74E−2
GO:0036094	small molecule binding	4.71E−4	2.90E−2
GO:0004054	arginine kinase activity	7.92E−4	3.95E−2
GO:0004869	cysteine-type endopeptidase inhibitor activity	7.92E−4	3.95E−2
GO:0005272	sodium channel activity	7.92E−4	3.95E−2
GO:0015078	hydrogen ion transmembrane transporter activity	9.95E−4	4.73E−2
**E2009-RD** **vs.** **E2010-RD (up-regulated)**			
–	–	–	–
**E2009-RD** **vs.** **E2010-RD (down-regulated)**			
GO:0004553	hydrolase activity. hydrolyzing O-glycosyl compounds	3.31E−12	1.23E−8
GO:0005515	protein binding	7.36E−7	2.37E−4
GO:0003676	nucleic acid binding	3.96E−6	8.21E−4
GO:0016817	hydrolase activity. acting on acid anhydrides	3.15E−5	4.52E−3
GO:0016462	pyrophosphatase activity	4.84E−5	6.15E−3
GO:0017111	nucleoside-triphosphatase activity	7.43E−5	8.94E−3
GO:0000166	nucleotide binding	8.98E−5	1.06E−2
GO:1901265	nucleoside phosphate binding	9.17E−5	1.06E−2
GO:0004022	alcohol dehydrogenase (NAD) activity	2.72E−4	3.02E−2
GO:0016491	oxidoreductase activity	3.59E−4	3.73E−2

The comparison of quiescent and diapaused eggs exposed to H-RD showed an increase in the redox cell control, cellulase and hydrolase activities and nucleic acid synthesis in down-regulated genes. The Fisher’s enrichment test performed with these two latter comparisons showed that more functions were activated in quiescent eggs compared with the diapaused eggs. Variation in hatching observed between the eggs of a single population could provide greater adaptive flexibility (Jones, Tylka & Perry, 1998). For example, a proportion of eggs could even require a second period of stimulation by RD to hatch in the second season of potato production in *G. rostochiensis* (Hominick, Forrest & Evans, 1985). This strategy helps to reduce competition between J2s for feeding sites and the persistence of the population in the field (Jones, Tylka & Perry, 1998).

Cellulase activity was up-regulated in the diapaused eggs. This could reflect the low levels of these genes in water treatment (as it has been seen in the comparison between both kinds of eggs with the same water treatment). Atkinson, Taylor & Fowler (1987) found that mechanically freed J2s of *G. rostochiensis* showed a partial increase in the nucleolus diameter of the dorsal pharyngeal gland cells when suspended in water but the J2s needed to be placed in PRD to achieve a nucleolus size similar to J2s hatched by stimulation with PRD. Palomares-Rius et al. (2012) did not find an increase in the nucleolus size in forced hatched diapaused juveniles after their exposure to RD. However, the gene expression of one cellulase gene showed a slight increase in expression in diapaused eggs hydrated and soaked for 24 h in potato root diffusate in comparison to only hydrated eggs and similar expression was seen with quiescent eggs (Palomares-Rius et al., 2012). Transport function related to the nervous system was up-regulated in diapaused eggs when eggs soaked in water or H-RD was compared. Other activities such as phosphorylation in quiescent hydrated eggs and nucleotide binding activity in H-RD soaked quiescent eggs were also up-regulated. These activities indicate metabolism activation in preparation for hatching.

### CLICK analysis of the differentially expressed genes

CLICK clustering analysis showed 5 major patterns of expression for differentially expressed genes and 32 genes were not included in these clusters (Fig. 2). Cluster 1 has 1,670 genes showing general up-regulated expression in quiescent eggs and mainly down-regulation in diapaused eggs, more so in hydrated than in H-RD-soaked diapaused eggs. Cluster 2 has 783 genes mainly up-regulated in diapaused eggs, particularly in the hydrated diapaused eggs. Cluster 3 has 242 genes mainly up-regulated in H-RD soaked quiescent eggs. Cluster 4 has 90 genes mainly genes up-regulated in hydrated quiescent and diapaused eggs. Cluster 5 has 32 genes and they were mainly down-regulated in hydrated quiescent eggs.

The functions differentially affected using Fisher’s enrichment analysis of Blast2GO annotations with this clustering, are shown in Tables S1 and S2. Only cluster 1 and 3 showed enrichment in functional terms in relation to the whole microarray. Cluster 1 was enriched in mainly hydrolase and binding activities and in transport activities, functions related to the activity of effectors and the activation of movement and perception of environmental signals inside the egg. Functions enriched in Cluster 3 were related to transmembrane transport activities. The other clusters did not show any particular enrichment function associated with them. The Fisher’s enrichment test performed with CLICK clusters showed major functions related to the gene expression in the different eggs (Table 6). Transport function was more expressed in cluster 1 and 3 with different genes expressed in each cluster. Cellulases were over-expressed in diapaused eggs are not influenced in the quiescent eggs in Cluster 1.

**Table 6:** Fisher enrichment test (FDR < 0.05) performed in Blast2GO v.2.7.0 for the different CLICK groups formed with the differentially expressed genes.

GO-ID	Term	*P*-value	FDR
**Cluster 1**			
GO:0016798	hydrolase activity, acting on glycosyl bonds	9.16E−12	1.16E−15
GO:0004553	hydrolase activity, hydrolyzing O-glycosyl compounds	2.55E−11	6.47E−15
GO:0008810	cellulase activity	2.35E07	1.19E−10
GO:0022891	substrate-specific transmembrane transporter activity	7.47E−07	4.74E−10
GO:0022838	substrate-specific channel activity	1.68E−06	1.45E−09
GO:0022803	passive transmembrane transporter activity	1.68E−06	1.71E−09
GO:0015267	channel activity	1.68E−06	1.71E−09
GO:0022892	substrate-specific transporter activity	5.99E−06	6.84E−09
GO:0015075	ion transmembrane transporter activity	9.27E−06	1.32E−08
GO:0022857	transmembrane transporter activity	9.27E−06	1.40E−08
GO:0005216	ion channel activity	9.27E−06	1.41E−08
GO:0005215	transporter activity	2.72E−05	5.42E−08
GO:0001871	pattern binding	2.72E−05	5.52E−08
GO:0030247	polysaccharide binding	2.72E−05	5.52E−08
GO:0030246	carbohydrate binding	1.95E−04	4.20E−07
GO:0015077	monovalent inorganic cation transmembrane transporter activity	7.90E−03	2.61E−05
GO:0043499	eukaryotic cell surface binding	1.96E−02	6.95E05
GO:0022834	ligand-gated channel activity	4.40E−02	1.90E−04
GO:0015276	ligand-gated ion channel activity	4.40E−02	1.90E−04
**Cluster 2**			
–	–	–	–
**Cluster 3**			
GO:0015291	secondary active transmembrane transporter activity	2.06E−08	1.63E−04
GO:0022804	active transmembrane transporter activity	9.35E−06	3.69E−02
**Cluster 4**			
–	–	–	–
**Cluster 5**			
–	–	–	–

### Quantitative RT-PCR analysis

Six genes that were differentially expressed in both microarray experiments were selected for confirmation by quantitative real-time RT-PCR. These results are shown in Table 1. Patterns of expression correlated well between the microarray and the quantitative RT-PCR.

## Conclusions

Quiescent and diapaused eggs of *G. pallida* respond in fundamentally different ways to hydration or exposure to root diffusate, as judged by gene analysis. Many unique genes were activated in the population of diapaused eggs. Genes associated with transport activity were activated in both quiescent and diapaused eggs, but the specific genes involved differed significantly between the two populations. Hydrated quiescent and diapaused eggs were markedly different indicating differences in adaptation for long-term survival. This study has taken into account a broad analysis of genes from the existing genome sequence of *G. pallida*.

## Supplemental Information

10.7717/peerj.1654/supp-1 Table S1 Microarray genes annotated using Blast2Go (v.2.7.0)

10.7717/peerj.1654/supp-2 Table S2 Genes differentially expressed between quiescent eggs soaked in water and soaked in water and exposed to tomato root diffusate (RD)

10.7717/peerj.1654/supp-3 Table S3 Genes differentially expressed between diapaused eggs soaked in water and soaked in water and exposed to tomato root diffusate (RD)

10.7717/peerj.1654/supp-4 Table S4 Genes differentially expressed between quiescent and diapaused eggs soaked in water

10.7717/peerj.1654/supp-5 Table S5 Genes differentially expressed between quiescent and diapaused eggs soaked in water and exposed to tomato root diffusate (RD)

10.7717/peerj.1654/supp-6 Table S6 Common genes differentially up-regulated between quiescent and diapaused eggs soaked in water eggs soaked in water and soaked in water and exposed to tomato root diffusate (RD)

10.7717/peerj.1654/supp-7 Table S7 Common genes differentially down-regulated between quiescent and diapaused eggs soaked in water eggs soaked in water and soaked in water and exposed to tomato root diffusate (RD)

10.7717/peerj.1654/supp-8 Table S8 Common genes differentially contra-regulated between quiescent and diapaused eggs soaked in water eggs soaked in water and soaked in water and exposed to tomato root diffusate (RD)

10.7717/peerj.1654/supp-9 Table S9 Common genes differentially up-regulated between quiescent and diapaused eggs soaked in water eggs and between quiescent and diapaused eggs soaked in water eggs soaked in water and soaked in water and exposed to tomato root diffusate (RD)

10.7717/peerj.1654/supp-10 Table S10 Common genes differentially down-regulated between quiescent and diapaused eggs soaked in water eggs and between quiescent and diapaused eggs soaked in water eggs soaked in water and soaked in water and exposed to tomato root diffusate (RD)

10.7717/peerj.1654/supp-11 Table S11 Common genes differentially contra-regulated between quiescent and diapaused eggs soaked in water eggs and between quiescent and diapaused eggs soaked in water eggs soaked in water and soaked in water and exposed to tomato root diffusate (RD)

10.7717/peerj.1654/supp-12 Table S12 Unique genes differentially up-regulated between quiescent eggs soaked in water and soaked in water and exposed to tomato root diffusate (RD)

10.7717/peerj.1654/supp-13 Table S13 Unique genes differentially up-regulated between diapaused eggs soaked in water and soaked in water and exposed to tomato root diffusate (RD)

10.7717/peerj.1654/supp-14 Table S14 Unique genes differentially up-regulated between quiescent eggs soaked in water and soaked in water and exposed to tomato root diffusate (RD)

10.7717/peerj.1654/supp-15 Table S15 Unique genes differentially up-regulated between diapaused eggs soaked in water and soaked in water and exposed to tomato root diffusate (RD)

10.7717/peerj.1654/supp-16 Table S16 Fisher enrichment test (FDR < 0.05) performed in Blast2GO v.2.7.0 for the different comparisons in the studied microarray samples. RD, Tomato root diffusate

10.7717/peerj.1654/supp-17 Figure S1 Schematic of treatments for *G. pallida* used in this study Samples consisted of cyst populations with eggs in quiescent state (E2009) and cyst populations with the majority of eggs in diapaused stage and some percentage in quiescent stage (approximately 10%)
Cysts were hydrated in water for 5 days and cysts were obtained for RNA extraction. The water was removed from the remaining cysts and tomato root diffusate (RD) was added for 4 days, after cysts were used for RNA extraction.

10.7717/peerj.1654/supp-18 Figure S2 (A) Venn diagram comparing differentially expressed genes between quiescent eggs when they were soaked in water and soaked in water and exposed to tomato root diffusate (RD) and diapaused eggs when they were soaked in water and soaked in water and exposed to RD. (B) Venn diagram comparing differentially expressed genes between quiescent and diapaused eggs when they were soaked in water and soaked in water and exposed to RD

10.7717/peerj.1654/supp-19 Figure S3 Molecular functions of genes differentially expressed using Blast2Go (v. 2.7.0): E2009HO, quiescent eggs soaked in water; E2010H2O, diapaused eggs soaked in water; E2009RD, quiescent eggs soaked in water and exposed to tomato root diffusate (RD); E2010RD, quiescent eggs soaked in water and exposed to RD. Down, down-regulated in comparison to the first treatment; up, up-regulated in comparison to the first treatment

## Additional Information and Declarations

### Microarray Data Deposition

The following information was supplied regarding the deposition of microarray data: ArrayExpress accession E-MTAB-3824.

### Data Availability

The following information was supplied regarding data availability: The data is provided in Tables S1–S16.
