# Supplementary material for: Gene expression changes in diapause or quiescent potato cyst nematode, Globodera pallida, eggs after hydration or exposure to tomato root diffusate
Source: PeerJ. 2016 Feb 4;4:e1654. doi: 10.7717/peerj.1654 (PMC4748719; doi:10.7717/peerj.1654)
Supplement: Table S16 — RD, Tomato root diffusate [file peerj-04-1654-s016.docx]

**Table S12.** Fisher enrichment test (FDR < 0.05) performed in Blast2GO v.2.7.0 for the different comparisons in the studied microarray samples. RD: Tomato root diffusate.

| **GO-ID** | **Term** | **P-value** | **FDR** | |
| --- | --- | --- | --- | --- |
| **E2009-H2O *vs* E2010-RD (UP-regulated)** | |  |  | |
| GO:0015291 | secondary active transmembrane transporter activity | 2.78E-7 | 2.19E-3 | |
| **E2009-H2O vs E2009-RD (DOWN-regulated)** | |  |  | |
| - | - | - | - | |
| **E2010-H2O *vs* E2010-RD (UP-regulated)** | |  |  | |
| GO:0008810 | cellulase activity | 9.74E-12 | 7.68E-8 | |
| GO:0004553 | hydrolase activity. hydrolyzing O-glycosyl compounds | 2.09E-9 | 6.70E-6 | |
| GO:0016798 | hydrolase activity. acting on glycosyl bonds | 2.55E-9 | 6.70E-6 | |
| GO:0030246 | carbohydrate binding | 1.50E-6 | 1.97E-3 | |
| GO:0001871 | pattern binding | 7.13E-6 | 4.59E-3 | |
| GO:0030247 | polysaccharide binding | 7.12E-6 | 4.59E-3 | |
| GO:0005488 | binding | 9.29E-6 | 5.23E-3 | |
| GO:0005515 | protein binding | 1.13E-5 | 5.45E-3 | |
| GO:0005215 | transporter activity | 1.16E-5 | 5.46E-3 | |
| GO:0004550 | nucleoside diphosphate kinase activity | 1.88E-5 | 6.75E-3 | |
| GO:0003676 | nucleic acid binding | 2.99E-5 | 9.44E-3 | |
| GO:0022891 | substrate-specific transmembrane transporter activity | 4.73E-5 | 1.42E-2 | |
| GO:0022857 | transmembrane transporter activity | 9.73E-5 | 2.02E-2 | |
| GO:0022892 | substrate-specific transporter activity | 1.05E-4 | 2.07E-2 | |
| GO:0043499 | eukaryotic cell surface binding | 1.81E-4 | 3.03E-2 | |
| GO:0016787 | hydrolase activity | 2.45E-4 | 3.88E-2 | |
| GO:0004022 | alcohol dehydrogenase (NAD) activity | 3.55E-4 | 4.99E-2 | |
| **E2010-H2O *vs* E2010-RD (DOWN-regulated)** | |  |  | |
| - | - | - | - | |
| **E2009-H2O *vs* E2010-H2O (UP-regulated)** | |  |  | |
| - | - | - | - | |
| **E2009-H2O *vs* E2010-H2O (DOWN-regulated)** | |  |  | |
| GO:0004553 | hydrolase activity. hydrolyzing O-glycosyl compounds | 2.35E-15 | | 4.63E-12 |
| GO:0016798 | hydrolase activity. acting on glycosyl bonds | 3.40E-15 | | 6.28E-12 |
| GO:0005488 | binding | 3.68E-14 | | 3.62E-11 |
| GO:0005515 | protein binding | 8.22E-13 | | 6.48E-10 |
| GO:0022891 | substrate-specific transmembrane transporter activity | 2.75E-11 | | 1.67E-8 |
| GO:0003676 | nucleic acid binding | 7.01E-11 | | 3.25E-8 |
| GO:0008810 | cellulase activity | 7.95E-11 | | 3.48E-8 |
| GO:0022838 | substrate-specific channel activity | 1.05E-10 | | 4.09E-8 |
| GO:0097159 | organic cyclic compound binding | 1.10E-10 | | 4.09E-8 |
| GO:1901363 | heterocyclic compound binding | 1.11E-10 | | 4.09E-8 |
| GO:0022803 | passive transmembrane transporter activity | 1.24E-10 | | 4.09E-8 |
| GO:0015267 | channel activity | 1.24E-10 | | 4.09E-8 |
| GO:0005216 | ion channel activity | 2.75E-10 | | 8.35E-8 |
| GO:0015075 | ion transmembrane transporter activity | 3.18E-10 | | 9.29E-8 |
| GO:0022857 | transmembrane transporter activity | 1.05E-9 | | 2.85E-7 |
| GO:0022892 | substrate-specific transporter activity | 2.23E-9 | | 5.50E-7 |
| GO:0001871 | pattern binding | 4.21E-8 | | 8.51E-6 |
| GO:0030247 | polysaccharide binding | 4.21E-8 | | 8.51E-6 |
| GO:0005215 | transporter activity | 8.36E-8 | | 1.65E-5 |
| GO:0030246 | carbohydrate binding | 2.76E-7 | | 5.07E-5 |
| GO:0016917 | GABA receptor activity | 1.22E-5 | | 1.46E-3 |
| GO:0015077 | monovalent inorganic cation transmembrane transporter activity | 1.39E-5 | | 1.63E-3 |
| GO:0022834 | ligand-gated channel activity | 2.50E-5 | | 2.63E-3 |
| GO:0015276 | ligand-gated ion channel activity | 2.50E-5 | | 2.63E-3 |
| GO:0005230 | extracellular ligand-gated ion channel activity | 3.62E-5 | | 3.66E-3 |
| GO:0043499 | eukaryotic cell surface binding | 5.82E-5 | | 5.40E-3 |
| GO:0003723 | RNA binding | 1.83E-4 | | 1.46E-2 |
| GO:0004563 | beta-N-acetylhexosaminidase activity | 2.07E-4 | | 1.47E-2 |
| GO:0004550 | nucleoside diphosphate kinase activity | 2.07E-4 | | 1.47E-2 |
| GO:0043027 | cysteine-type endopeptidase inhibitor activity involved in apoptotic process | 2.07E-4 | | 1.47E-2 |
| GO:0003677 | DNA binding | 4.41E-4 | | 2.74E-2 |
| GO:0036094 | small molecule binding | 4.71E-4 | | 2.90E-2 |
| GO:0022890 | inorganic cation transmembrane transporter activity | 6.96E-4 | | 3.95E-2 |
| GO:0008324 | cation transmembrane transporter activity | 7.32E-4 | | 3.95E-2 |
| GO:0015929 | hexosaminidase activity | 7.92E-4 | | 3.95E-2 |
| GO:0004054 | arginine kinase activity | 7.92E-4 | | 3.95E-2 |
| GO:0004869 | cysteine-type endopeptidase inhibitor activity | 7.92E-4 | | 3.95E-2 |
| GO:0005272 | sodium channel activity | 7.92E-4 | | 3.95E-2 |
| GO:0015078 | hydrogen ion transmembrane transporter activity | 9.95E-4 | | 4.73E-2 |
| **E2009-RD *vs* E2010-RD (UP-regulated)** | |  |  | |
| - | - | - | - | |
| **E2009-RD *vs* E2010-RD (DOWN-regulated)** | |  |  | |
| GO:0004553 | hydrolase activity. hydrolyzing O-glycosyl compounds | 3.31E-12 | | 1.23E-8 |
| GO:0008810 | cellulase activity | 4.69E-12 | | 1.23E-8 |
| GO:0001871 | pattern binding | 1.96E-10 | | 3.09E-7 |
| GO:0030247 | polysaccharide binding | 1.96E-10 | | 3.09E-7 |
| GO:1901363 | heterocyclic compound binding | 2.80E-8 | | 1.48E-5 |
| GO:0097159 | organic cyclic compound binding | 2.81E-8 | | 1.48E-5 |
| GO:0030246 | carbohydrate binding | 3.89E-8 | | 1.92E-5 |
| GO:0005515 | protein binding | 7.36E-7 | | 2.37E-4 |
| GO:0003676 | nucleic acid binding | 3.96E-6 | | 8.21E-4 |
| GO:0005488 | binding | 2.89E-5 | | 4.22E-3 |
| GO:0016817 | hydrolase activity. acting on acid anhydrides | 3.15E-5 | | 4.52E-3 |
| GO:0016462 | pyrophosphatase activity | 4.84E-5 | | 6.15E-3 |
| GO:0016818 | hydrolase activity. acting on acid anhydrides. in phosphorus-containing anhydrides | 5.06E-5 | | 6.33E-3 |
| GO:0017111 | nucleoside-triphosphatase activity | 7.43E-5 | | 8.94E-3 |
| GO:0000166 | nucleotide binding | 8.98E-5 | | 1.06E-2 |
| GO:1901265 | nucleoside phosphate binding | 9.17E-5 | | 1.06E-2 |
| GO:0004022 | alcohol dehydrogenase (NAD) activity | 2.72E-4 | | 3.02E-2 |
| GO:0016491 | oxidoreductase activity | 3.59E-4 | | 3.73E-2 |
