# Supplementary figures and images for: Gene expression changes in diapause or quiescent potato cyst nematode, Globodera pallida, eggs after hydration or exposure to tomato root diffusate
Source: PeerJ. 2016 Feb 4;4:e1654. doi: 10.7717/peerj.1654 (PMC4748719; doi:10.7717/peerj.1654)

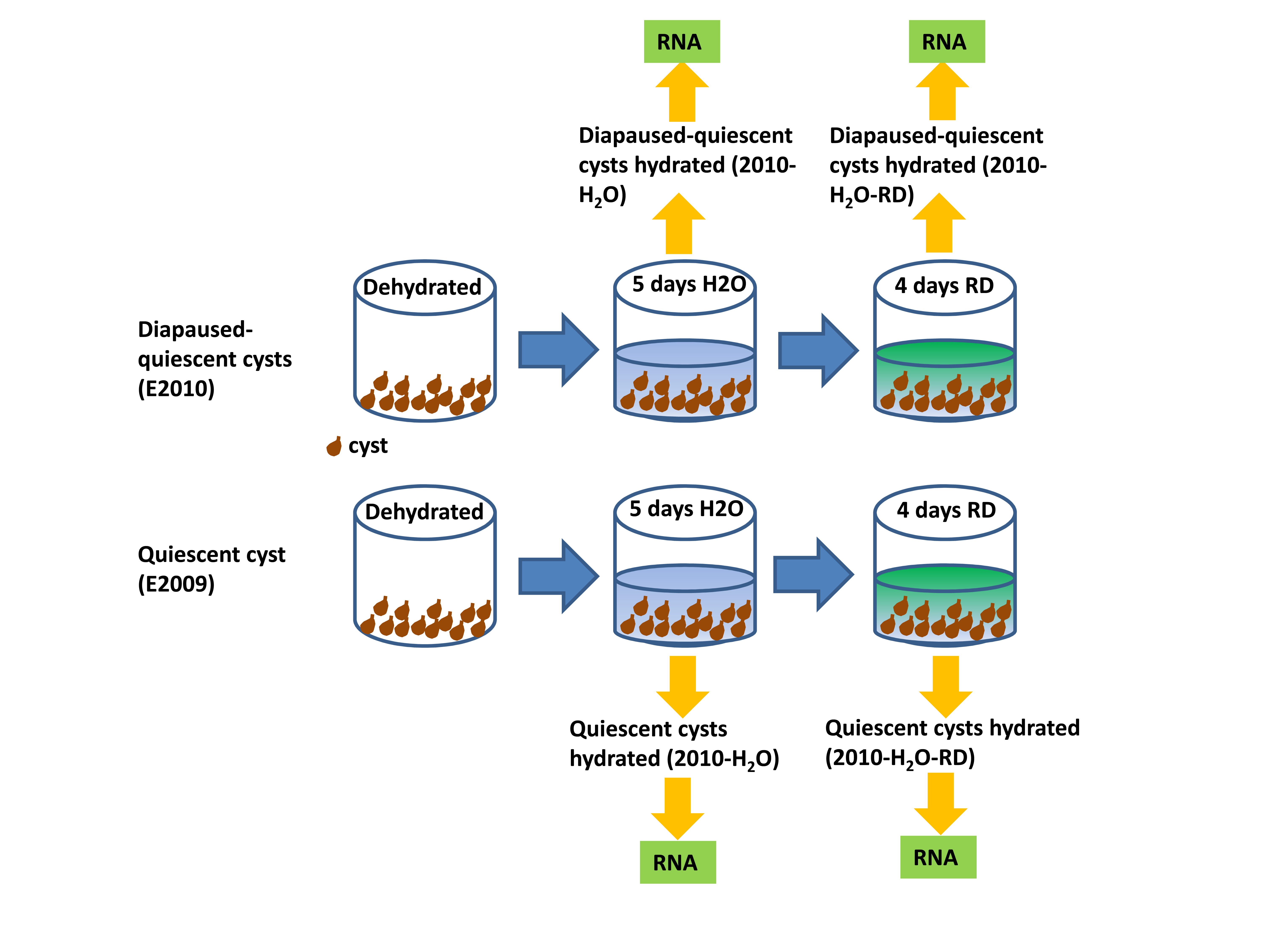

Supplement: Figure S1 — Samples consisted of cyst populations with eggs in quiescent state (E2009) and cyst populations with the majority of eggs in diapaused stage and some percentage in quiescent stage (approximately 10%) Cysts were hydrated in water for 5 days and cysts were obtained for RNA extraction. The water was removed from the remaining cysts and tomato root diffusate (RD) was added for 4 days, after cysts were used for RNA extraction. [file peerj-04-1654-s017.png]

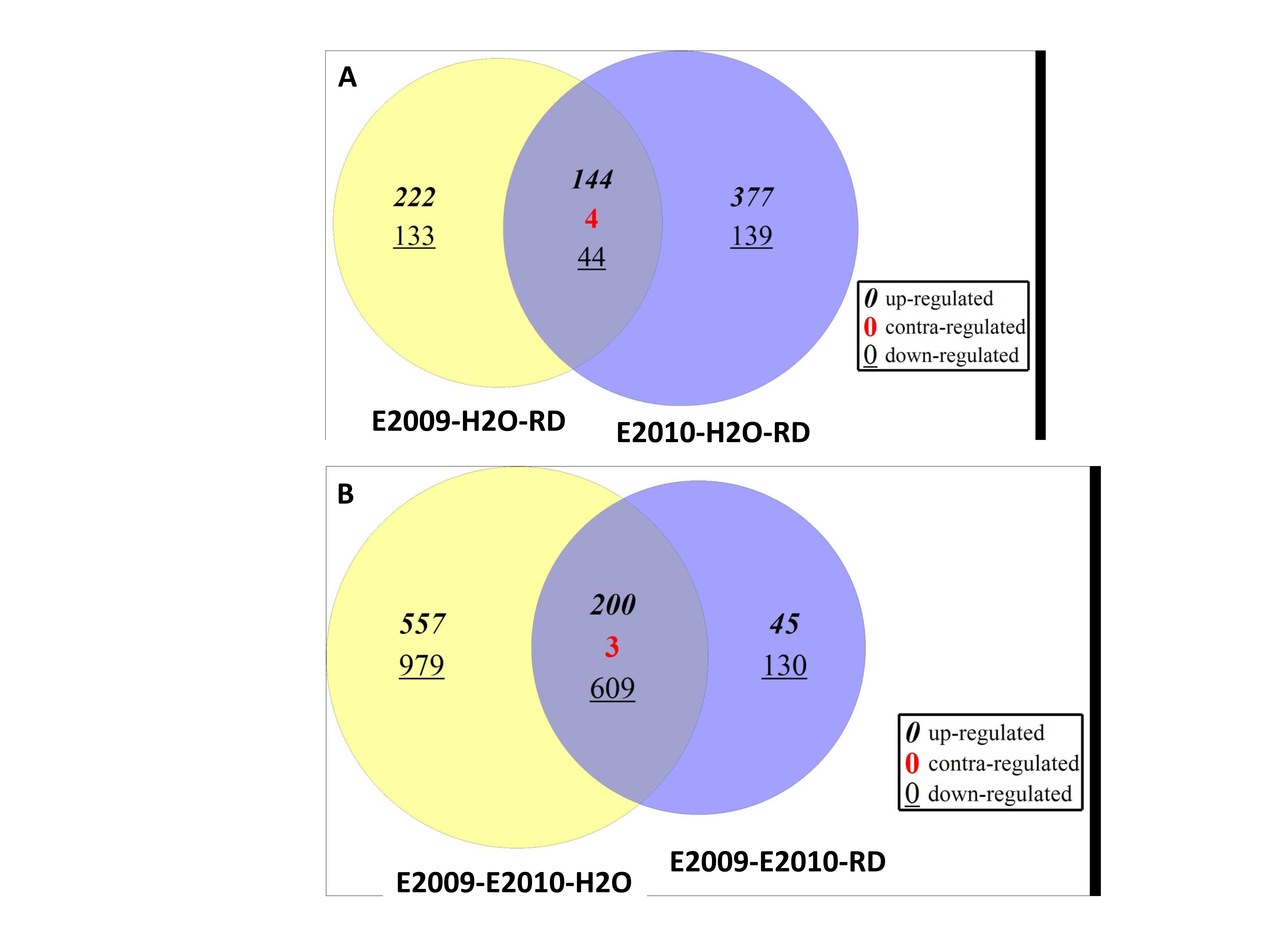

Supplement: Figure S2 [file peerj-04-1654-s018.png]

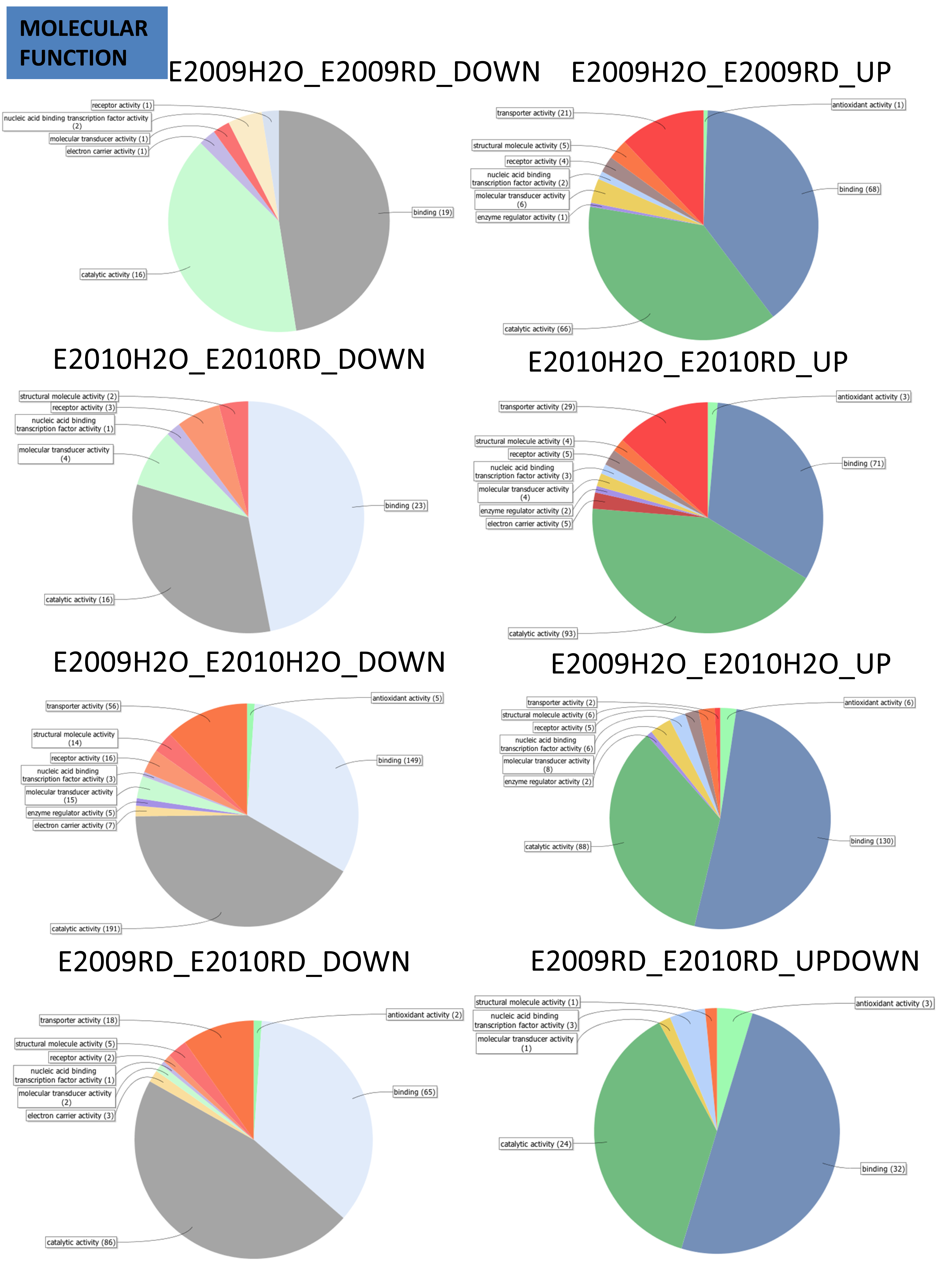

Supplement: Figure S3 [file peerj-04-1654-s019.png]
